# Label-Free High-Throughput Screening of CYP3A4 Inhibitors Using Acoustic Ejection Mass Spectrometry

**DOI:** 10.1021/acs.analchem.5c08059

**Published:** 2026-04-17

**Authors:** Mary Ashley Rimmer, Jingheng Wang, Tharindu A. Ranathunge, Zhe Shi, Yong Li, DongGeun Lee, Sergio C. Chai, Wenwei Lin, Nathaniel R. Twarog, Aseem Z. Ansari, Anang A. Shelat, Brandon M. Young, Taosheng Chen, Lei Yang

**Affiliations:** 1 Analytical Technologies Center, Department of Chemical Biology and Therapeutics, 541707 St. Jude Children’s Research Hospital, Memphis, Tennessee 38105, United States; 2 Department of Chemical Biology and Therapeutics, St. Jude Children’s Research Hospital, Memphis, Tennessee 38105, United States; 3 Compound Management, Department of Chemical Biology and Therapeutics, St. Jude Children’s Research Hospital, Memphis, Tennessee 38105, United States; 4 Medicinal Chemistry Center, Department of Chemical Biology and Therapeutics, St. Jude Children’s Research Hospital, Memphis, Tennessee 38105, United States; 5 Lead Discovery Informatics, Department of Chemical Biology and Therapeutics, St. Jude Children’s Research Hospital, Memphis, Tennessee 38105, United States

## Abstract

Label-free high-throughput screening (HTS) technologies have greatly accelerated early drug discovery by allowing direct measurement of biochemical activity without the need for chemical labels. Techniques like MALDI-TOF, RapidFire, desorption electrospray ionization, and acoustic-mist ionization mass spectrometry have shown strong analytical performance. More recently, acoustic droplet ejection mass spectrometry (AEMS) has emerged as a next-generation platform capable of analyzing over a thousand data points per hour. In this study, we thoroughly evaluated an AEMS workflow using cytochrome P450 3A4 (CYP3A4) as a model enzyme. Screening a library of 9702 compounds, this work represents the first successful label-free HTS campaign for CYP3A4 inhibitors in a 384-well format using AEMS. The result using nifedipine as a substrate was comparable to the widely used P450-Glo luminescence assay but with fewer inhibitor hits identifications. To further compare and parse differences between the methods and the substrates, 819 hits were retested using AEMS with luciferin isopropyl acetal, the same substrate used in the luminescence assay. The findings confirmed that both the assay method and the substrate influence which inhibitors were detected. Overall, this study established AEMS as a robust and reliable tool for CYP3A4 inhibitor screening. It offers high analytical precision, fewer false positives, compatibility with multiple substrates, and direct detection of metabolites, providing a practical way to validate hits and improve confidence in early drug discovery efforts.

## Graphical abstract

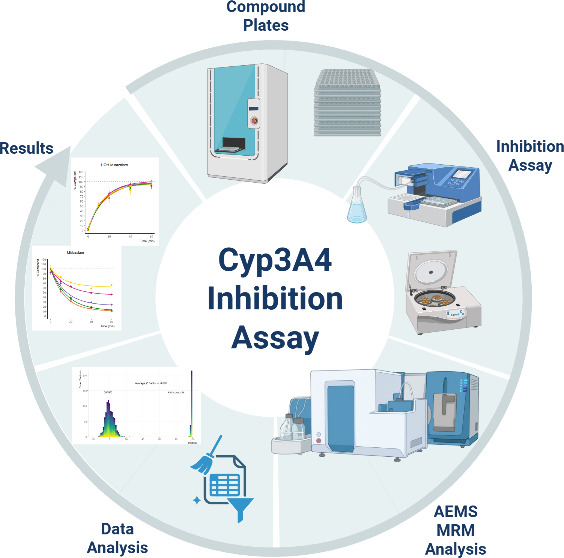

## Introduction

Cytochrome P450 3A4 (CYP3A4) is one of the most influential enzymes in human drug metabolism, contributing to the clearance of nearly half of all marketed pharmaceuticals.^1,2^ Its broad substrate promiscuity allows it to metabolize structurally diverse xenobiotics, ranging from small molecule therapeutics to dietary constituents and environmental compounds.^3^ Because of this high level of involvement in clearing therapeutics from the liver, coadministering CYP3A inhibitors with targeted treatments can increase the therapeutic efficacy of treatment. However, broadly inhibiting CYP3A enzymes can have negative systemic effects. Consequently, reliable identification of specific CYP3A4 inhibitors is essential for minimizing metabolic impacts, guiding medicinal chemistry decisions, and reducing attrition during later development stages.^4^

High-throughput screening (HTS) of CYP3A4 inhibitors has traditionally relied on fluorescence- or luminescence-based assays because of their compatibility with microplate formats,^1,5–10^ allowing them to process thousands of compounds per day. In these assays, CYP3A4 activity is monitored using reporter substrates, such as 7-benzyloxy-4-trifluoromethylcoumarin for fluorescence^11,12^ or Luciferin isopropyl acetal (IPA) for luminescence,^13,14^ that generate measurable optical signals when metabolized by the enzyme. The P450-Glo luminescence assay has become widely adopted due to its high sensitivity, broad dynamic range, and straightforward workflow based on luciferase detection chemistry.^14,15^ These methods enable rapid identification of inhibitors and have supported numerous large-scale screening campaigns across pharmaceutical and academic settings. However, despite their efficiency, optical assays are inherently vulnerable to signal interference from test compounds, including auto luminescence, quenching, inner-filter effects, and direct interaction with the luciferase detection system.^16–18^ Such interference can affect the behaviors of the compounds differently among assays, especially when dealing with chemically diverse libraries.^19^

Mass spectrometry (MS) is a powerful alternate technology due to its capability to directly detect both substrates and metabolites with high analytical specificity in a label-free manner. Unlike optical assays, MS-based platforms do not rely on optical reporters and are largely unaffected by compound-related signal artifacts. Advances in high-throughput MS have substantially accelerated analysis speed.^20^ Technologies such as MALDI-TOF MS support HTS by enabling rapid analysis of thousands of samples.^21,22^ More recent developments, such as the Agilent RapidFire system which automated solid-phase extraction with MS detection, reduce cycle times to 2.5 s per sample.^23^ Furthermore, emerging ionization approaches including segmented-flow electrospray ionization,^24,25^ desorption electrospray ionization (DESI),^26,27^ and acoustic-mist ionization MS (AMI-MS) have pushed throughput to approximately multiple samples per second,^28,29^ making them viable for ultrahigh-throughput screening (uHTS). Among these innovations, acoustic ejection mass spectrometry (AEMS) has gained prominence.^30^ By combining acoustic droplet ejection with an open-port interface (OPI) for electrospray ionization mass spectrometry (ESI-MS), AEMS enables nanoliter-scale sample delivery at rates comparable to optical assays while retaining the selectivity and robustness of MS.^31,32^ Its minimal sample consumption, negligible analytical preparation, and compatibility with diverse assay matrices make AEMS uniquely suited to streamline biochemical screening workflows.^33–41^

The rising importance of CYP3A inhibition profiling in drug discovery heightens the value of such technological advances. Drug substrates such as midazolam and physiological substrates such as testosterone, nifedipine, are commonly used to quantify CYP3A4 activity, with the formation of their oxidative metabolites measured by liquid chromatography–mass spectrometry (LC-MS).^42–44^ However, CYP3A4 possesses an unusually flexible active site, which leads to substrate-dependent differences in inhibitor potency.^43,45–51^ Relying on a single probe substrate increases the risk of overlooking mechanistically relevant inhibitors or mischaracterizing their spotency.^52^ Thus, screening platforms need to not only achieve high analytical sensitivity and throughput but also allow for the biochemical relevance necessary for substrate-informed interpretation. Label-free MS approaches, particularly AEMS, are well positioned to fulfill these requirements. By enabling rapid, direct quantification of probe substrate turnover without optical interference, AEMS meets the speed requirements for uHTS. This capability is increasingly crucial as therapeutic discovery programs seek to reduce false positives, streamline hit identification, and improve the reliability of early decision-making.

In this study, we provide the first comprehensive evaluation of AEMS for CYP3A4 inhibitor screening and compare its performance directly with established luminescence-based assays. We also implement a parallel probe-substrate strategy to assess how substrate choice influences inhibitor identification, addressing a long-standing challenge in CYP3A4 characterization. Together, our results demonstrate that AEMS is a robust and reliable platform for CYP3A4 inhibitor screening. It delivers high analytical precision, reduces false positives, supports multiple probe substrates, and enables direct metabolite detection, offering a practical and confident route for validating hits in early drug discovery.

## Experimental Section

### Materials

Drug-like compounds were cherry-picked from the St. Jude collection,^53^ representing 53% of the entire library based on scaffold diversity (2D molecular fingerprint-based clustering) and key structural properties, including the absence of structural alerts (PAINS), lipophilicity (ALogP 3–5), H-bond donors ≤ 5, H-bond donors plus acceptors ≥ 3, and molecular weight between 300–500 Da (Figure 1). Nifedipine, Ritonavir, Ammonium Floride, and DMSO were purchased from Sigma (St. Louis, MO, cat. no. N7634, SML0491, 338869, D8418). Dehydronifedipine and 1-OH Midazolam were obtained from Cayman Chemical (Ann Arbor, MI, cat. no. 23229 and 10385), Midazolam from U.S. Pharmacopeia (North Bethesda, MD, cat. no. 1443599), and Luciferin IPA from Promega (Madison, WI; cat. no. V9002). Warfarin was sourced from Fluka Chemical Corp. (Ronkonkoma, NY, cat. no. A2250), Ketoconazole from Abovchem (San Diego, CA, cat. no. AC513267), and SJ000362065 and Z56791366 from Enamine (Monmouth Junction, NJ, cat. no. Z56792718 and Z56791366). Luciferin isopropyl ester was synthesized in-house (Supporting Information Figure S1). Compound purity was verified by UPLC-MS.

**Figure 1:**
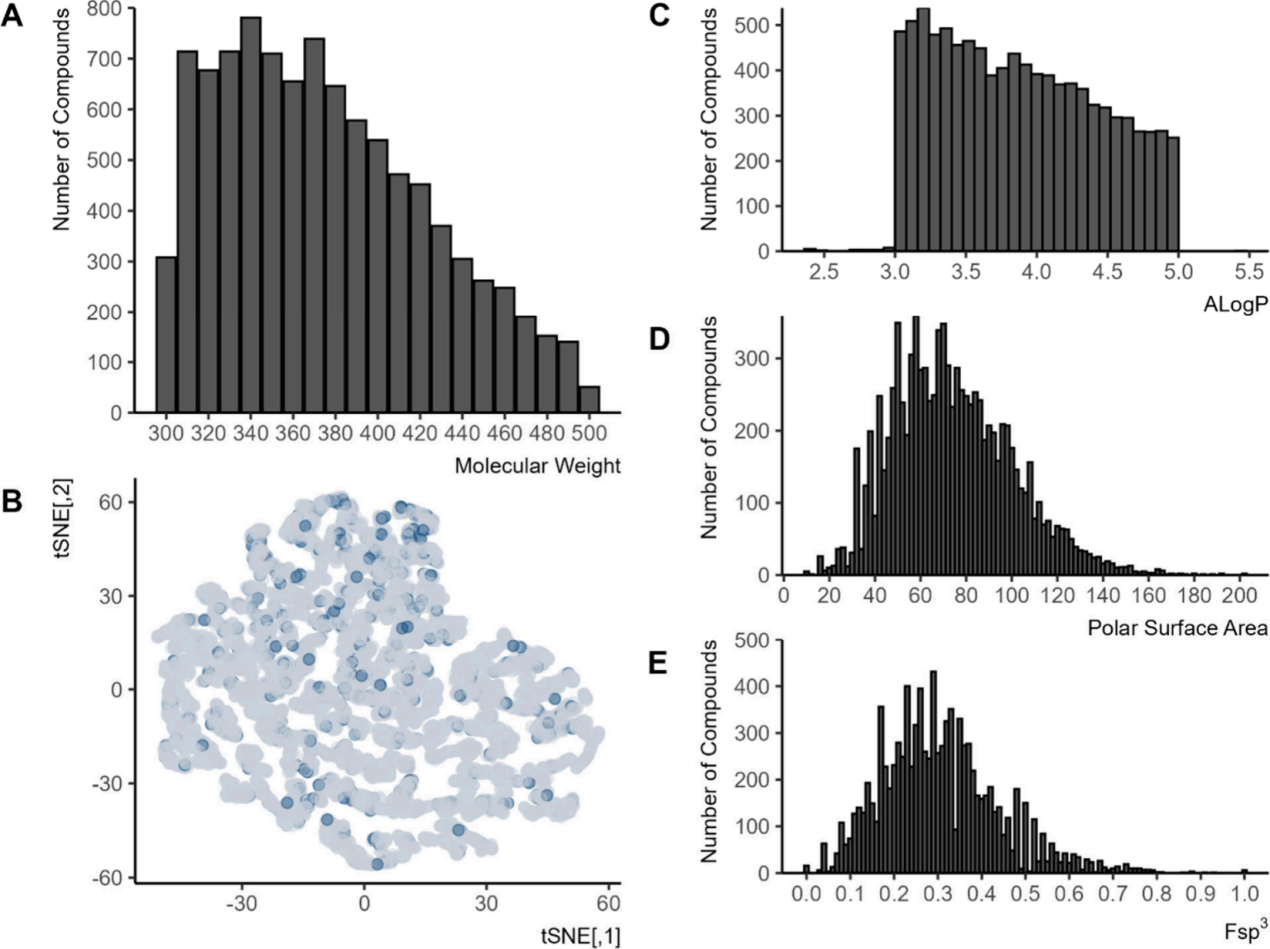
Histogram of library compounds (*n* = 9702). (A) The distribution of molecular weights. (B) Visualization of all 4 properties, as distributed across the library compounds. Dark blue points are inhibitors as defined by the AEMS_Nif assay (≥80% inhibition). (C) AlogP of the library compounds. (D) Polar surface area. (E) Fsp^3^.

LC-MS grade methanol and acetonitrile were acquired from Fisher Scientific (Waltham, MA). Milli-Q water (Burlington, MA) was used for reagent preparation. Analytical 96-well and 384-well microtiter plates were purchased from Corning (product 3363 and 3657), and Echo-MS qualified 384-well polypropylene plates (PP) and cyclic olefin copolymer (COC) low dead volume (LDV) microplates were obtained from Beckman Coulter Life Sciences (San Jose, CA; cat. nos. C74290 and LP-0200).

### Preparation of Compound Plates

Time-Course Study: Nifedipine and Midazolam stocks were prepared in a 2-fold serial dilution (0.5 mM to 8 mM in DMSO) and aliquoted into 384-well PP plates. From the master plate, 20 nL was transferred to assay plates yielding 0.5–8 μM final concentrations in 20 μL reactions. All compound stocks and DMSO controls were dispensed in technical triplicate using an Echo acoustic dispenser 655 (Beckman Coulter Inc., Indianapolis, Indiana), with a final DMSO concentration of 0.1%.

Dose–Response Curves: Ketoconazole, Ritonavir, SJ000362065, and Z56791366 were tested in 3-fold serial dilutions prepared from 40 mM DMSO stocks. From each master plate, 30 nL was transferred to assay plates to generate 0.34 nM to 60 μM final concentrations in 20 μL reactions. Nifedipine or Luciferin IPA stocks (2 or 3 mM in DMSO) were plated separately, and 20 nL was transferred to achieve final substrate concentrations of 2 or 3 μM. All assays were run in triplicate with a final DMSO concentration of 0.25%.

Single-Concentration HTS: The screening compounds were transferred to assay plates to yield a final concentration of 10 μM in 20 μL reactions (columns 3–12, 15–24). Ketoconazole (30 μM final concentration) was served as the positive control in columns 1 and 13, while DMSO was served as the negative control in columns 2 and 14. Nifedipine or Luciferin IPA (20 nL) was added to all wells for final concentrations of 2 or 3 μM, respectively. All transfers were performed using the Echo acoustic dispenser, with a final DMSO concentration of 0.2%.

### CYP3A4 Inhibition by AEMS

AEMS-based CYP3A4 inhibition assays using Nifedipine and Luciferin IPA as the substrates (AEMS_NIF and AEMS_LUC) were performed in a 384-well assay format. An in-house library of 9702 drug-like compounds (including redundancies; Figure 1) was screened at 10 μM for CYP3A4 inhibition using the AEMS_NIF assay. In addition, a subset of 819 prescreened inhibitor candidates was further evaluated at 10 μM using the AEMS_LUC assay.

Recombinant human CYP3A4 Supersome (Corning, cat. no. 456202), which contained P450 oxidoreductase (POR) and Cytochrome *b*_5_, was used as the enzyme source. Substrates and test compounds were predispensed into the assay plates, followed by the addition of 10 μL 2× enzyme–substrate mixture and 10 μL of 2× NADPH regeneration system (Promega, Madison, WI, cat. no. V9510). The final concentrations of reaction components were as follows: 10 nM CYP3A4, 2 μM Nifedipine or 3 μM Luciferin IPA, 0.34 nM to 60 μM of the test compound, and 1× NADPH regeneration system in the 100 mM potassium phosphate buffer (pH 7.4). The final DMSO concentration was up to 0.25%. Wells containing 30 μM ketoconazole were used as the positive control (100% inhibition), whereas wells with DMSO only were used as the negative control (0% inhibition). The workflow was presented in Figure 2.

**Figure 2:**
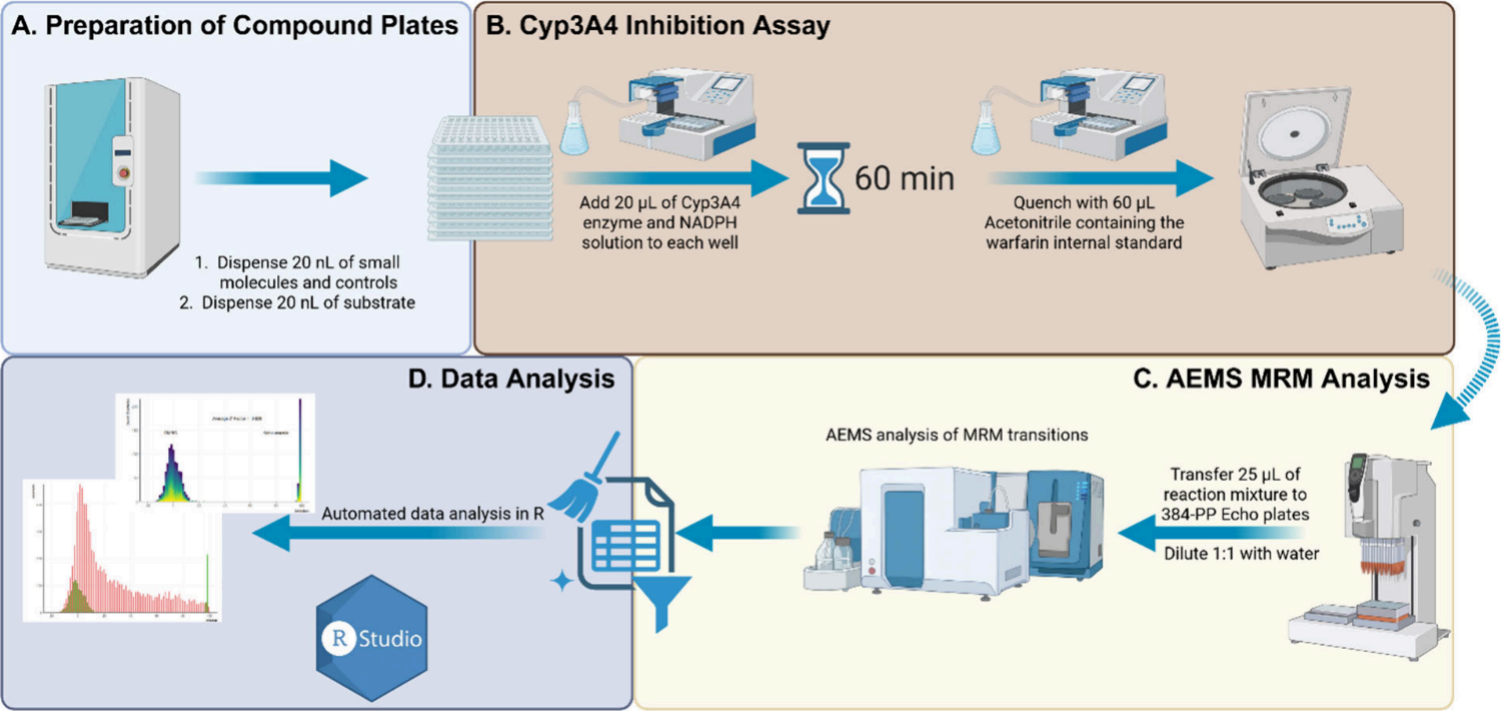
CYP3A4 inhibitor screening workflow. (A) Preparation of compound plates. The screening workflow began by using an Echo liquid dispenser to transfer 20 nL of small-molecule compounds, controls, and an additional 20 nL of substrate into 384-well plates. (B) CYP3A4 inhibition assay. 20 μL of a CYP3A4 enzyme and NADPH mixture was added to the preloaded plates, followed by a 60 min incubation. Reactions were stopped by adding 60 μL of acetonitrile containing warfarin as the internal standard, after which the plates were sealed and centrifuged at 4000 rpm for 20 min. (C) AEMS MRM analysis. A total of 25 μL of the resulting supernatant was then transferred to fresh 384-well polypropylene Echo plates using an automated liquid handler and mixed with an equal volume of water. Plates were centrifuged at 2000 rpm for 5 min, shaken at 1350 rpm for 30 s, and subsequently analyzed on the AEMS system. (D) Data analysis. Data processing and quality evaluation were performed using in-house automated R workflows for cleanup and analysis.

The assay plates were incubated at room temperature for 60 min, after that the reaction was quenched with 60 μL of acetonitrile containing 800 ng/mL warfarin as an internal standard for MS analysis. Plates were then centrifuged at 4000 rpm for 20 min, and 25 μL of the resulting supernatant was transferred to fresh polypropylene (PP) plates and diluted with an equal volume of Milli-Q water. The plates were subsequently stored at −80 °C until analysis. Prior to measurement, the frozen plates were thawed at room temperature, centrifuged at 2,000 rpm for 5 min, and shaken at 1,350 rpm for 30 s to remove gas bubbles and to ensure a uniform fluid meniscus. These procedures provided consistent sample preparation and minimized variability, enabling accurate quantification of CYP3A4 inhibition by test compounds using the AEMS platform.

### AEMS System

Mass spectrometric detection was performed on an AB Sciex Triple Quad 6500+ system (AB Sciex, Concord, ON, Canada) equipped with an AEMS acoustic autosampler and controlled by Sciex OS Analytics Software v3.1.0, operating in positive-ion electrospray ionization (ESI) and multiple reaction monitoring (MRM) modes. Ten nL of samples were acoustically ejected from 384-well polypropylene plates into the open port interface at a carrier solvent and transferred via capillary to the OptiFlow Turbo V ion source, with acoustic firing performed in normal mode using a 2000 ms interval with a standard peak type. Optimized ESI source parameters included GS1 at 90 psi, GS2 at 50 psi, curtain gas at 35 psi, collision gas (CAD) at 9 units, a source temperature of 500 °C, and an ion spray voltage of 5500 V. MRM transitions and AEMS operational details for Nifedipine, Dehydronifedipine, Luciferin IPA, Luciferin isopropyl ester, and Warfarin are listed in Supplemental Table S1. Raw data (.wiff files) were processed using Sciex OS, with peak integration thresholds set to a minimum signal-to-noise ratio of 2, a minimum peak height of 100 counts, and retention-time windows of at least 0.02 min.

### Data Analysis

All data was analyzed using in-house R scripts. For AEMS-based CYP3A4 inhibition assays, MS data was first processed using Sciex OS. The MS peak area data was extracted and reported as the percent conversion of substrate to product (eq 1), for both AEMS_NIF and AEMS_LUC assays. The substrate/product pairs monitored were Nifedipine/Dehydronifedipine for AEMS_NIF, and Luciferin IPA/Luciferin isopropyl ester for AEMS_LUC.

1 $$\mathrm{\% Conversion}=\frac{\mathrm{Area product}}{\mathrm{Area substrate}+\mathrm{Area product}}$$

For comparison with LUMI-LUC assays, CYP3A4 inhibition was also calculated (eq 2) using wells containing 30 μM of ketoconazole as the positive control (100% inhibition) and DMSO wells were the negative control (0% inhibition).

2 $$\mathrm{\% Inhibition}=100-,100\times \frac{\mathrm{\% Conversion sample}-\mathrm{\% Conversion Ketoconazole}}{\mathrm{\% Conversion DMSO}-\mathrm{\% Conversion Ketoconazole}}$$

IC_50_ values of known CYP3A4 inhibitors were calculated from dose–response curves using the same method as previously described.^46^

Assay performance was evaluated using the Z′-factor,^54^

3 $$Z^{\prime }\mathrm{‐factor}=1-\left[3\times \left(\sigma_{p}+\sigma_{n}\right)\right]/\left|\mu_{p}-\mu_{n}\right|$$

where μ_p_ and σ_p_ were the mean and standard deviation of the positive control; μ_n_ and σ_n_ are the mean and standard deviation of the negative control.

A variant of Stochastic Neighbor Embedding, t-SNE, was applied to visualize the molecular information (Figure 1B). This allows mapping of high-dimensional data into two or three dimensions without crowding, revealing multiscale structure across complex manifolds.^55^

### R Analysis

All data analysis was performed in R, an open-source programming and data science environment,^56,57^ and the RStudio IDE,^58^ and visualized using the ggplot2 package.^59^

## Results and Discussion

### Setup of CYP3A4 AEMS Assay

Starting from the reaction conditions established for the gold-standard luminescence P450-Glo assay with Luciferin IPA as substrate (LUMI_LUC), we systematically optimized key parameters to adapt the workflow for AEMS. Enzyme concentration, incubation time, internal standard levels, quenching solvent type, and quenching volume were each evaluated across a range of conditions, and the most robust settings were selected for subsequent experiments (data not shown). These optimizations ensured sufficient substrate turnover while maintaining reproducibility and linearity in a high-throughput format. In parallel, AEMS instrument parameters were fine-tuned based on the mass spectral responses of representative CYP3A4 substrates, Nifedipine, Midazolam, and Luciferin IPA, and their respective metabolites. Factors such as carrier solvent, ion additives, MRM transitions, and tandem MS voltages were adjusted iteratively to maximize signal intensity and minimize background interference. Final optimized conditions are provided in the Supporting Information Table S1, collectively supporting a sensitive and reliable high-throughput AEMS workflow (Figure 2).

To identify the most suitable substrate, we evaluated Lovastatin,^60^ Nifedipine,^43^ Dextromethorphan,^51^ and Midazolam^43^ in a time-course study using 2-fold dilutions from 0.5 to 8 μM. Both parent compounds and their well-characterized metabolites were assessed under reaction conditions comparable to the LUMI_LUC assay. Nifedipine and Midazolam showed strong metabolic turnover, with less than 20% of the parent remaining after 60 min (Figure 3A), while Lovastatin and Dextromethorphan underperformed (data not shown). Given its widespread use in CYP3A4 inhibitor assays and accessibility, Nifedipine was chosen as the substrate for the preliminary high-throughput AEMS screening.^42–44^ Furthermore, time-course analysis with Nifedipine and its oxidated metabolite, Dehydronifedipine, confirmed that 2 μM provided an optimal balance of substrate consumption and product formation while reaching saturation over the incubation period (Figure 3A).

**Figure 3:**
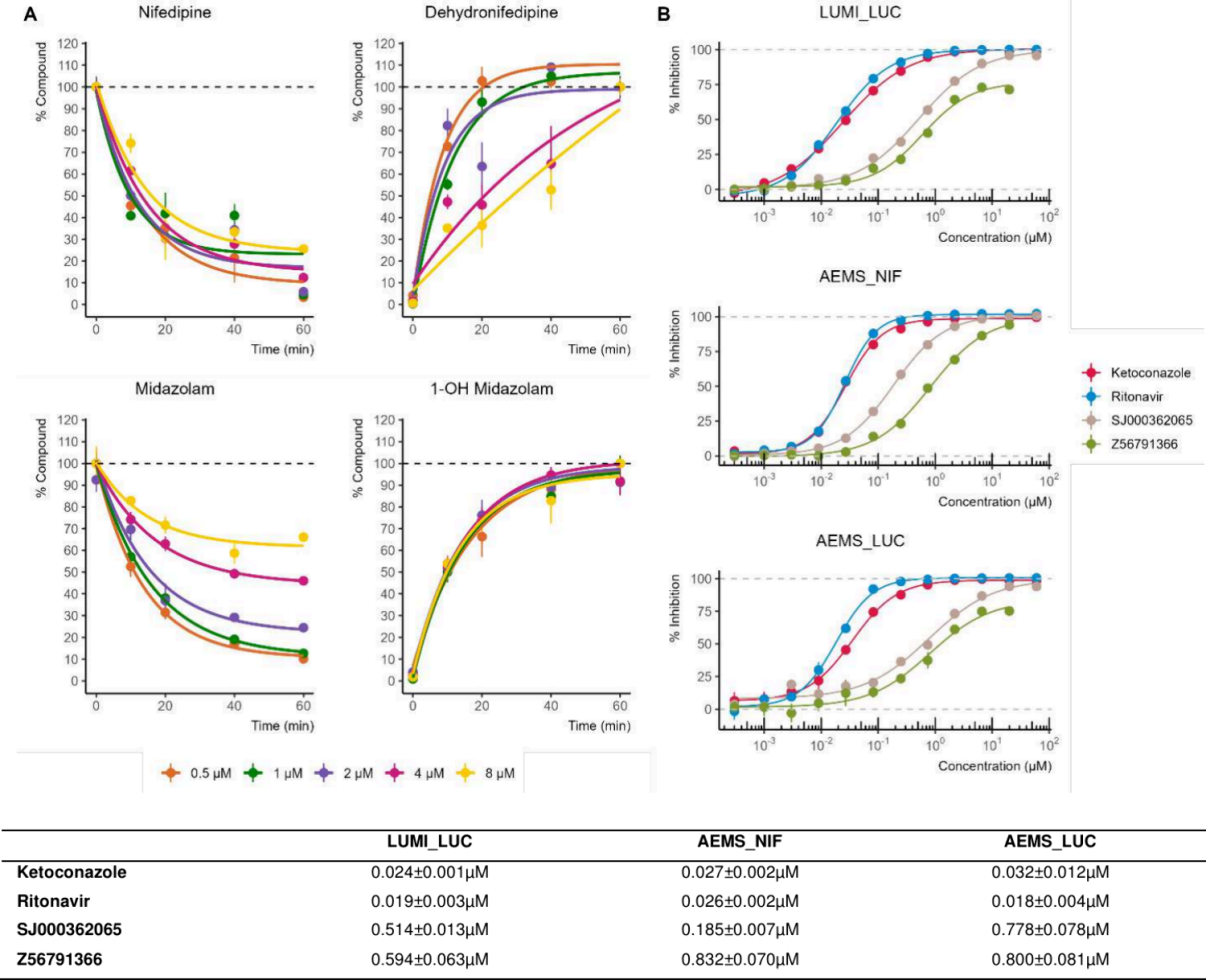
Inhibition profiles. (A) Time-course profiles of nifedipine and midazolam metabolism, showing substrate depletion and metabolite formation over the incubation period. (B) Dose–response curves for four well-characterized CYP3A4 inhibitors, ketoconazole, ritonavir, SJ000362065, and Z56791366, measured across three assay formats, LUMI_LUC, AEMS_NIF, and AEMS_LUC, illustrating cross-platform consistency in inhibitory potency.

For consistency with the LUMI_LUC assay, Luciferin IPA was also evaluated in the AEMS format at the same substrate concentration recommended by the manufacturer (3 μM). The Luciferin isopropyl ester metabolite showed significantly stronger MS signal than the final product of the LUMI-LUC assay, D-luciferin, producing more than a 2-fold increase in response (data not shown). Consequently, Luciferin isopropyl ester was selected as the product to monitor. Due to the substrate limited availability, only the 819 hits identified by both AEMS_NIF and LUMI_LUC were further assessed using AEMS_LUC.

Four benchmark CYP3A4 inhibitors, Ketoconazole, Ritonavir, SJ000362065, and Z56791366, were profiled in dose–response format across AEMS_NIF, AEMS_LUC, and LUMI_LUC. All compounds showed comparable IC_50_ values across platforms, supporting the reliability of AEMS for quantitative CYP3A4 assessment (Figure 3B). Ketoconazole and ritonavir exhibited consistent sub-50 nM IC_50_ potency in all assays. SJ000362065 showed submicromolar IC_50_ inhibition, with IC_50_ values ranging from 0.185 ± 0.007 μM (AEMS_NIF) to 0.778 ± 0.078 μM (AEMS_LUC). Z56791366 demonstrated moderate potency, with IC_50_ values of 0.594 ± 0.063 μM in LUMI_LUC, 0.800 ± 0.081 μM in AEMS_LUC, and 0.832 ± 0.070 μM in AEMS_NIF. Together, these data show strong cross-platform agreement, with only minor differences driven by substrate and detection modality.

### Preliminary Screening Validation with AEMS_NIF

As part of the AEMS_NIF approach validation, the performance of the internal standard (I.S.) was closely monitored to ensure consistency across the entire screening set. A total of 9702 compounds were evaluated for the CYP3A4 inhibition across 31 assay plates. Warfarin was chosen as the internal standard due to its reliable ionization and consistent mass spectrometric response at the designated concentration and ejection volume. Across all plates, the warfarin signal intensity consistently reached or exceeded 0.5 million counts, demonstrating robust detection and stable ionization throughout the runs. The plate-to-plate variation was tightly controlled, reflecting a high degree of operational reproducibility. The coefficient of variation (CV%) for warfarin across all plates ranged from 6% to 15%, well within acceptable limits for high-throughput MS-based assays. These results confirmed the analytical stability of the AEMS_NIF workflow and provided confidence in the reliability of the sample preparation process (Figure 4A).

**Figure 4:**
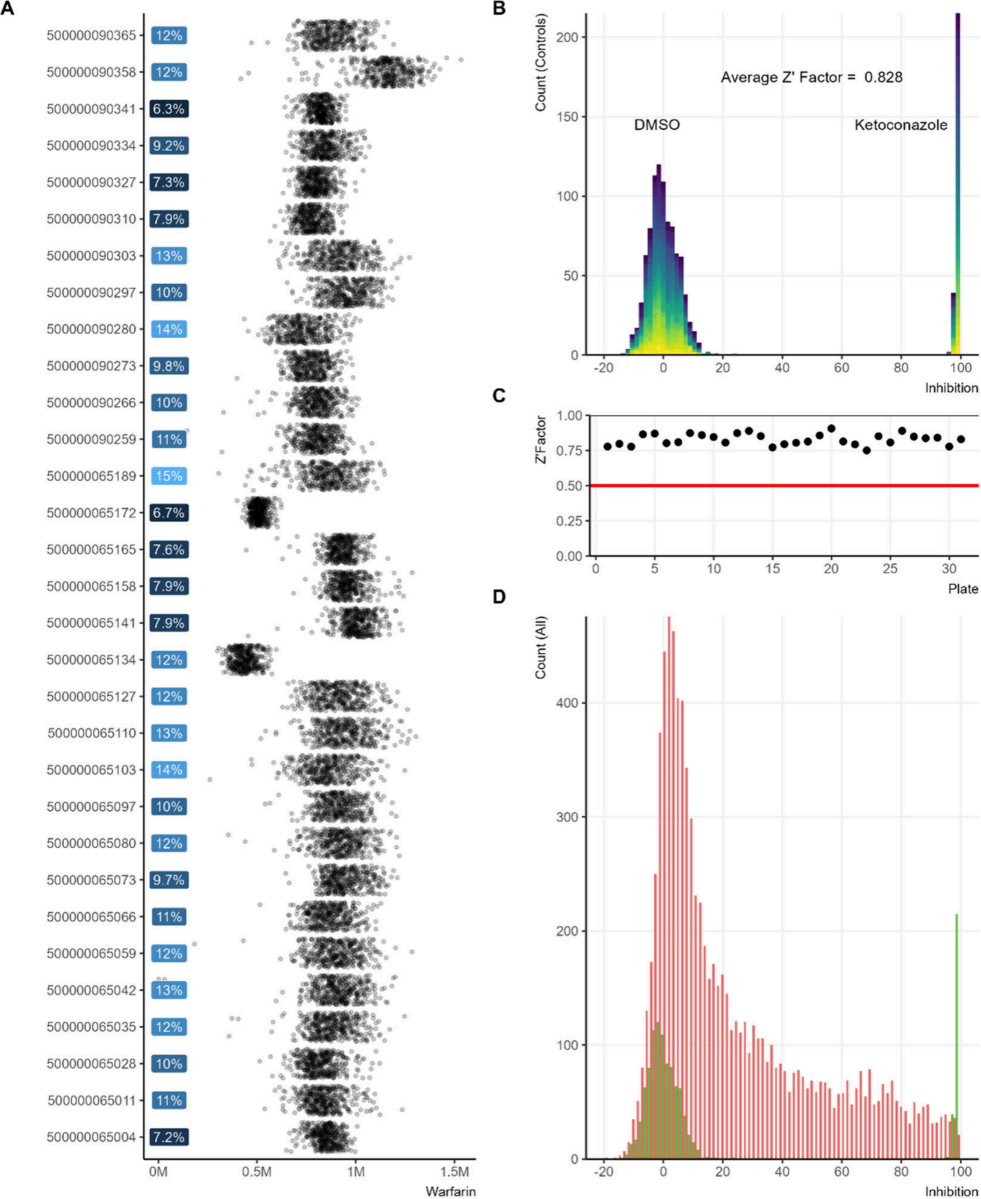
Evaluate the plate variation. (A) Dot plots represent the area of the internal standard for each of the 31 plates. Blue percentages are the CV of each respective plate, with the darker blue showing lower variation. (B) The control inhibition plotted vs the number of positive and negative control at each inhibition level. The clear spread between the positive and negative controls is mathematically demonstrated by the average Z′ factor of 0.828. (C) The Z′ factors for each plate. (D) The control inhibition (green) plotted along with the inhibition of each compound (red).

Assay performance was further validated by analyzing positive control wells (100% inhibition by Ketoconazole) and negative control wells (0% inhibition by DMSO) across all inhibition levels (Figure 4B). Both control groups displayed tight variation and a normal distribution centered around their expected theoretical values, demonstrating stable and well-behaved assay performance. The robustness and reproducibility of the workflow were further supported by an average Z′ factor of 0.828, indicative of excellent assay quality (Figure 4C).^54^ Z′ factors calculated for each individual plate consistently met high-performance thresholds, confirming that all aspects of the screening workflow, including liquid handling, bioassay execution, sample preparation, instrument acquisition, mass spectrometric readout, and data analysis, remained stable and reliable throughout the entire campaign. This comprehensive validation established the AEMS_NIF method as a highly reproducible and robust platform for high-throughput CYP3A4 inhibitor screening.

### Comparison of Inhibition Screening Methods

A total of 9702 drug-like compounds were screened using the AEMS_NIF method, and the resulting distribution of CYP3A4 inhibition responses was summarized in Figure 4D. In comparison, inhibition data obtained from the luminescence-based LUMI_LUC assay were sourced from a previously published data set containing 8,979 unique compounds.^61^ Across these two data sets, 7,360 compounds overlapped which provided a direct basis for cross-method analysis. Within this shared compound set, an 80% inhibition cutoff was applied to define active inhibitors. Using this threshold, AEMS_NIF identified 378 compounds as inhibitors, corresponding to approximately 5.2% of the pool, whereas LUMI_LUC detected 785 inhibitors, or roughly 10.7%. Among these, 350 compounds (4.8%) were consistently identified as inhibitors by both assays, reflecting a significant degree of concordance in detecting active compounds across independent assay platforms. Conversely, 28 compounds (0.4%) were identified solely by AEMS_NIF, while 435 compounds (5.9%) were flagged only by LUMI_LUC. The remaining 6,547 compounds (approximately 89%) were consistently classified as noninhibitors across both platforms, highlighting broad agreement for inactive compounds. Overall, inhibition values for the overlapping compounds exhibited strong correlation between the two detection platforms, with an R^2^ value of 0.785, suggesting that both assays capture similar inhibitory trends (Figure 5, Table 1).

**Figure 5:**
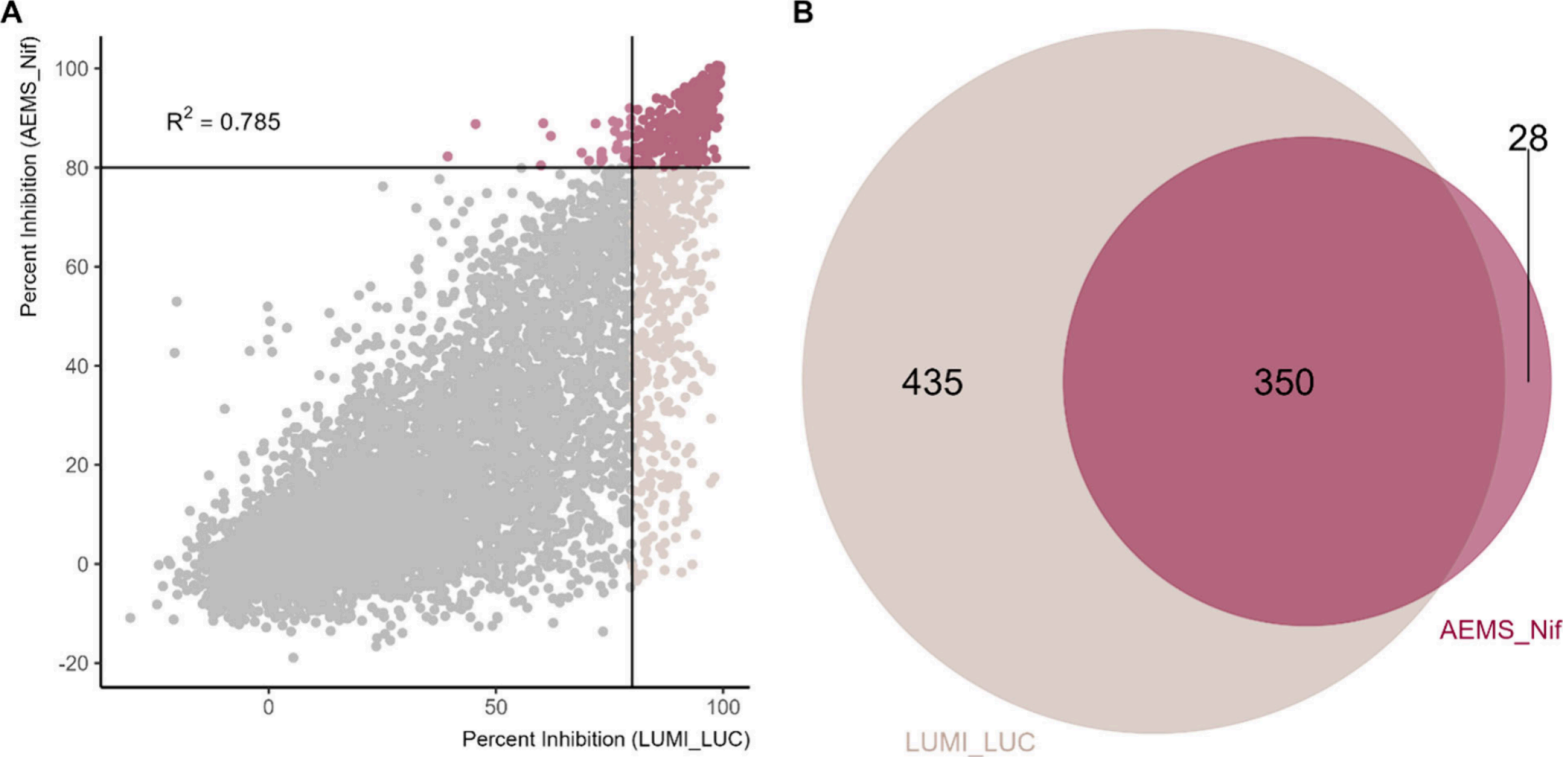
Scatter plot comparing inhibition percentages obtained from the two screening methods. (A) A total of 7360 compounds show strong correlation between methods (R^2^ = 0.785). (B) Primary screening resulted in 378 inhibitors (5.2%) in the AEMS_NIF assay and 785 inhibitors (11%) in the LUMI_LUC assay. A total of 350 inhibitors were shared between the two assays.

**Table 1:** Comparison of CYP3A4 Inhibitors Identified Using AEMS_NIF, AEMS_LUC, and LUMI_LUC Screening Approaches^a^

			Inhibitor						
	Total # of Compounds	Noninhibitor	LUMI_LUC only	AEMS_NIF only	AEMS_LUC only	LUMI_LUC & AEMS_NIF	AEMS	LUC	All approaches
Preliminary Screening									
LUMI_LUC	8979	8030 (89.4)	949 (10.6)						
AEMS_NIF	8808	8303 (94.3)		505 (5.7)					
LUMI_LUC ∩ AEMS_NIF	7360	6547 (89.0)	435 (5.9)	28 (0.4)		350 (4.8)			
819 Selective inhibitors from LUMI_LUC vs AEMS_NIF									
LUMI_LUC	819	35 (4.3)	784 (95.7)						
AEMS_NIF	819	441 (53.8)		378 (46.2)					
AEMS_LUC	819	416 (50.8)			403 (49.2)				
LUMI_LUC ∩ AEMS_NIF	819	7 (0.9)	434 (53.0)	28 (3.4)		350 (42.7)			
AEMS	819	335 (40.9)		81 (9.9)	106 (12.9)		297 (36.3)		
LUC	819	26 (3.2)	390 (47.6)		9 (1.1)			394 (48.1)	
All Approaches	819	7 (0.9)	328 (40.0)	19 (2.3)	0 (0)	62 (7.6)	9 (1.1)	106 (12.9)	288 (35.2)
484 Selective inhibitors from AEMS									
AEMS inhibitors	484			81 (16.7)	106 (21.9)		297 (61.4)		

a Data are presented as the number of compounds, with the percentage relative to each cohort shown in parentheses.

Interestingly, the number of inhibitors identified by AEMS_NIF (n = 28) but missed by LUMI_LUC was markedly lower than the number of inhibitors detected exclusively by LUMI_LUC (n = 435). This asymmetry provides important insight into the performance characteristics of each assay. It highlights both the strengths of AEMS_NIF as well as its method-dependent sensitivity relative to the luminescence assay. As widely reported,^19,62^ MS-based platforms avoid false-positive inhibition signals commonly caused by interference with luciferase or esterase,^18^ a known limitation of luminescence-based detection.^16,63^ In addition to detecting technological differences, the inhibitory effect of small molecules on CYP3A4 can vary depending on the specific substrate being metabolized. CYP3A4 possesses a large and flexible active site capable of accommodating multiple ligands simultaneously, which contributes to substrate-dependent variations in inhibition profiles.^45–48^

To determine whether discrepancies in inhibitor identification were driven more by the detection technology or by the choice of substrate, we selected 819 compounds that had been flagged as inhibitors by either AEMS_NIF or LUMI_LUC, along with several compounds that fell near the 80% inhibition cutoff. These compounds were re-evaluated using an AEMS assay that employed Luciferin IPA as the substrate. This orthogonal design provided a clear and systematic way to pinpoint the sources of variability in CYP3A4 inhibition results. By comparing MS and luminescence readouts while keeping the substrate constant, we were able to distinguish differences caused by the detection methods themselves from those reflecting true biochemical properties. This helped identify instances where luminescence signals were affected by compound interference or assay artifacts, as well as cases where both platforms showed strong agreement despite operating on different principles. In addition, assessing two distinct substrates using the same AEMS detection allowed us to probe CYP3A4’s well-known substrate-dependent inhibition behavior.^43,49,51^ This comparison revealed how subtle differences in substrate chemistry can shift inhibitor rankings or apparent potency.

Screening with the AEMS_LUC method revealed that of the 819 compounds, 403 (49.2%) were identified as inhibitors and 416 (50.8%) were classified as noninhibitors (Table 1). The inhibitor to noninhibitor ratio closely matched the distribution observed with the AEMS_NIF assay (46.2% vs 53.8%), demonstrating the consistency and reproducibility of the AEMS platform across different substrates. As observed previously, AEMS generally detected approximately half the number of inhibitors identified by the luminescent method, underscoring the difference in sensitivity and highlighting the lower false-positive rate inherent to MS-based detection. When comparing the two methods using the same substrate, Luciferin IPA, the AEMS_LUC assay identified 394 inhibitors (48.1%) that were also detected by the LUMI_LUC approach, demonstrating strong agreement under matched biochemical conditions. In addition, 9 compounds (1.1%) were uniquely detected as inhibitors by AEMS_LUC, while 390 inhibitors identified by the LUMI_LUC assay were not confirmed by MS. These observations support two important conclusions: first, methodological differences between detection platforms influence inhibitor identification, even when using the same substrate; second, the unique hits detected by luminescence alone highlight the risk of false-positive signals caused by interference with luciferase or esterase.

Further examination of substrate-dependent behavior revealed additional insights. Among 484 inhibitors identified by either AEMS_NIF or AEMS_LUC, 297 compounds (61.4%) were consistently identified as inhibitors across both substrates, demonstrating substantial agreement and reinforcing the reliability of AEMS for robust, interference-free detection. However, substrate-specific differences were observed: 81 compounds (16.7%) were exclusively identified when Nifedipine served as the CYP3A4 substrate, whereas 106 compounds (21.9%) were detected only with Luciferin IPA. These differences emphasize that, even with a highly reproducible mass-spectrometry readout, the inherent substrate-dependent activity of CYP3A4 can shape inhibitor profiles. The enzyme’s multiple binding sites influence both the enzyme, ligand binding affinity and the ligand’s conformational stability, explaining why different substrates reveal different inhibition patterns.^47,48^

## Conclusion

This study presented the first HTS campaign for CYP3A4 inhibitors using AEMS, introducing a label-free approach for biochemical assays. The sets of inhibitors identified by the AEMS_NIF and LUMI_LUC approaches were distinct. When we compared AEMS results with those from the widely used luminescence assay using the same substrate, Luciferin IPA, we found that AEMS identified roughly half as many inhibitors as the LUMI method. This difference does not indicate a loss of inhibitory activity but instead highlights a major advantage of MS-based detection: it avoided false-positive inhibition signals caused by optical interference or luciferase inhibition, which are common issues in luciferase-based systems. To further examine whether substrate choice contributed to the discrepancies, we evaluated inhibition profiles using AEMS with either Nifedipine or Luciferin IPA as the CYP3A4 substrate. Even under identical measurement technology, the two substrates yielded only 60% common inhibitor sets, reflecting the well-known substrate-dependent behavior of CYP3A4. Because CYP3A4 has a large and flexible active site capable of accommodating multiple binding orientations, inhibitor potency often varies with substrate, a principle clearly demonstrated here at high throughput.

Together, these results established AEMS as a robust and complementary platform for CYP3A4 inhibitor screening. The method offers excellent analytical precision, fewer false positives, compatibility with multiple substrates, and direct measurement of metabolite formation. The dual-substrate design further provides a practical way to separate true biochemical effects from detection artifacts, increasing confidence in hits confirmation and prioritization. As such, AEMS represents a powerful and scalable tool for accurate inhibitor discovery.

## Supplementary Material



## Notes

A comparison table of MRM transitions and AEMS parameters, the synthesis of Luciferase isopropyl ester, and the corresponding NMR spectra, chromatograms, and high-resolution mass spectra (PDF)
