# Supplementary material for: Label-Free High-Throughput Screening of CYP3A4 Inhibitors Using Acoustic Ejection Mass Spectrometry
Source: Anal Chem. 2026 Apr 17;98(17):12508–18. doi: 10.1021/acs.analchem.5c08059 (PMC13151951; doi:10.1021/acs.analchem.5c08059)
Supplement: Supplementary file 1 [file ac5c08059_si_001.pdf]

Supporting Information

Label-Free High-Throughput Screening of CYP3A4 Inhibitors Using Acoustic Ejection Mass Spectrometry

Mary Ashley Rimmer[1†], Jingheng Wang[2†], Tharindu A. Ranathunge[1, 3], Zhe Shi[4], Yong Li[1], DongGeun Lee[1], Sergio C. Chai[2], Wenwei Lin[2], Nathaniel R. Twarog[5], Aseem Z. Ansari[2], Anang A. Shelat[5], Brandon M. Young[4], Taosheng Chen[2], Lei Yang[1*]

[1] Analytical Technologies Center, Department of Chemical Biology and Therapeutics, St. Jude Children's Research Hospital, Memphis, Tennessee, 38105, USA

[2] Department of Chemical Biology and Therapeutics, St. Jude Children's Research Hospital, Memphis, Tennessee, 38105, USA

[3] Compound Management, Department of Chemical Biology and Therapeutics, St. Jude Children's Research Hospital, Memphis, Tennessee, 38105, USA

[4] Medicinal Chemistry Center, Department of Chemical Biology and Therapeutics, St. Jude Children's Research Hospital, Memphis, Tennessee, 38105, USA

[5] Lead Discovery Informatics, Department of Chemical Biology and Therapeutics, St. Jude Children's Research Hospital, Memphis, Tennessee, 38105, USA

[†]Co-first authors

*Corresponding author; e-mail: Lei.Yang@stjude.org

Table of Contents

Description	Page No.
**Table S1. Acoustic Ejection Mass Spectrometry Conditions Including Carrier Solvent, MRM Transitions, and MS Parameter Settings (DP, EP, CE, CXP).**	S3
**Synthesis of Luciferin Isopropyl Ester**	S4
**Figure S1. ^{1}H/^{13}C NMR Spectra and LCMS/HRMS Data for Luciferin Isopropyl Ester.**	S6,7

Table S1: Acoustic Ejection Mass Spectrometry Conditions Including Carrier Solvent, MRM Transitions, and MS Parameter Settings (DP, EP, CE, CXP).

Compound Name	Carrier Solvent	Precursor	Product Ion	DP (V)	EP (V)	CE (V)	CXP (V)
Nifedipine	100%Methanol with 1mM NH4F	347.1	315.1	60	10	13	10
Dehydronifedipine		345.1	284.2	100	10	40	18
Luciferin IPA	80:20 Acetonitrile:water with 1mM NH4F	367.2	247.1	50	10	26	17
Luciferin isopropyl ester		323.1	281.1	90	10	24	9
Warfarin	Applied with substrate	309.1	251.1	50	10	29	8

Synthesis of Luciferin Isopropyl Ester

General Methods and Materials. All reagents and solvents were obtained from commercially available sources and were used without further purification. (*S*)-2-(6-hydroxybenzo[d]thiazol-2-yl)-4,5-dihydrothiazole-4-carboxylic acid was obtained from Combi Blocks INC. (catalog #: QE-8258). Reactions were set up in air and carried out under nitrogen atmosphere. Purification was performed on the Waters purification LC/ UV/ELSD system. Column: Gemini -NX packed C18 50 mm × 30 mm, 5 μm. Collection: UV @ 214 nm and/or ELSD. Nuclear magnetic resonance (NMR) spectra were obtained on a Bruker NMR spectrometer at 500 MHz for ^{1}H-NMR spectra and 125 MHz for ^{13}C-NMR spectra. Chemical shifts (ppm) are reported relative to the solvent peak. Signals are designated as follows: s, singlet; d, doublet; dd, doublet of doublet; hept, heptet. Coupling constants *J* are expressed in Hertz. High resolution mass spectral data were obtained on a Waters Xevo Multi-reflecting Time-of-Flight mass spectrometer. The purity of final compounds was performed on an Acquity BEH C18 1.7 μm, 2.1 x 50 mm column (Waters Corporation, Milford, MA) using an Acquity ultra performance liquid chromatography (UPLC) system. The flow rate was 1 mL/min. The sample injection volume was 2 μL. The UPLC column was maintained at 63°C and the gradient program started at 90% A (0.1% formic acid in MilliQ H_2O), changed to 30% B (0.1% formic acid in Acetonitrile) in 0.2 min and increased to 95% B over 1.4 min, held for 0.35 minutes, then to 90% A over 0.05 minutes.

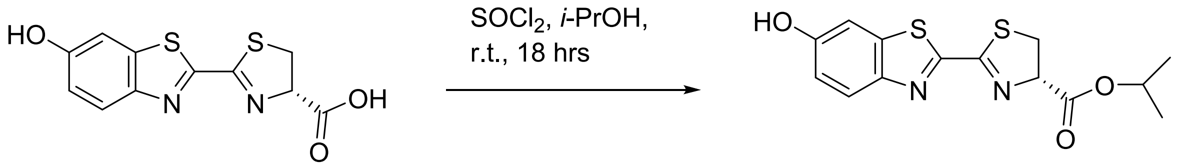

(*S*)-2-(6-hydroxybenzo[d]thiazol-2-yl)-4,5-dihydrothiazole-4-carboxylic acid (20 mg, 0.071 mmol) was added to a vial equipped with a stir bar, followed by isopropanol (1 mL). Thionyl chloride (10.4 μl, 0.14 mmol) was added into mixture. The reaction was stirred at room temperature overnight and then concentrated under vacuum. The crude product was purified on the Waters purification LC/ UV/ELSD system, and evaporations were carried out using a

TurboVap® LV evaporator to afford obtain isopropyl (S)-2-(6-hydroxybenzo[d]thiazol-2-yl)-4,5-dihydrothiazole-4-carboxylate (12 mg, 0.037 mmol, 52 % yield, Purity > 95%). ^{1}H NMR (500 MHz, DMSO-d_6) δ 10.25 (s, 1H), 7.97 (d, *J* = 8.9 Hz, 1H), 7.45 (d, *J* = 2.5 Hz, 1H), 7.07 (dd, *J* = 8.9, 2.4 Hz, 1H), 5.45 (dd, *J* = 9.8, 8.2 Hz, 1H), 5.01 (hept, *J* = 6.2 Hz, 1H), 3.79 (dd, *J* = 11.3, 9.8 Hz, 1H), 3.65 (dd, *J* = 11.3, 8.2 Hz, 1H), 1.26 (dd, *J* = 6.3, 2.1 Hz, 6H). ^{13}C NMR (126 MHz, DMSO-d_6) δ 169.80, 165.44, 157.92, 157.03, 146.70, 137.70, 125.39, 117.66, 107.28, 78.34, 69.43, 35.12, 21.97, 21.94. LCMS (m/z) $[M+H]^+$ = 323.4. HRMS: calcd for $C_{14}H_{14}N_2O_3S_2$ + H: 323.0519; found: 323.0521 (Figure S1).

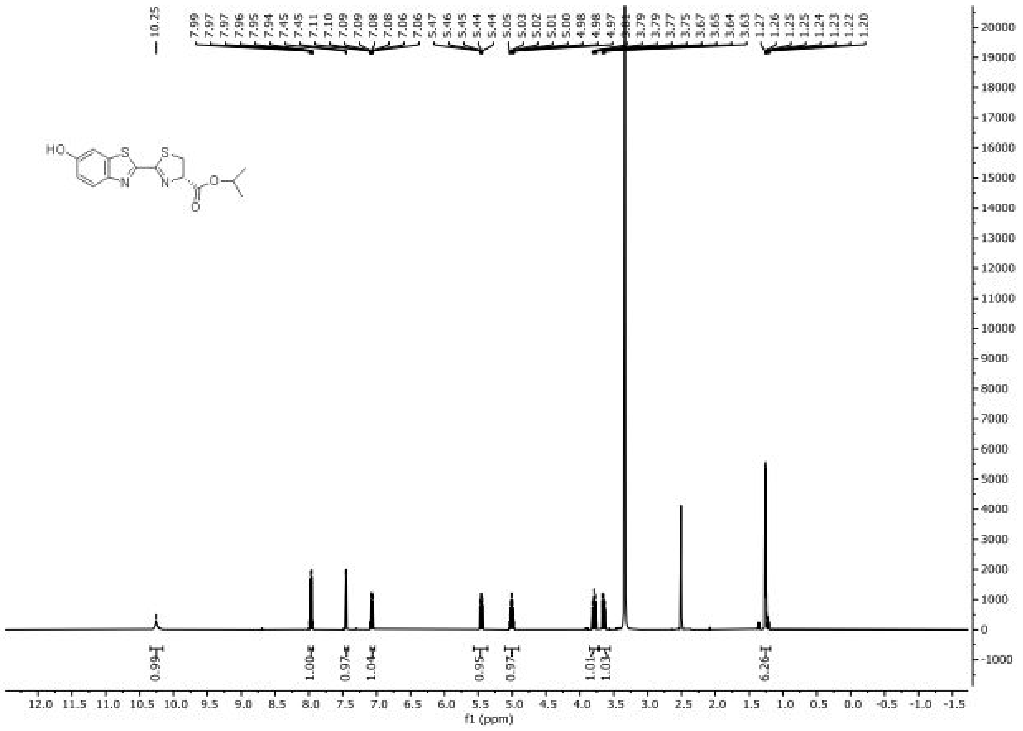
10.25
7.99 7.97 7.97 7.96 7.95 7.94 7.45 7.45 7.45 7.11 7.10 7.09 7.09 7.08 7.08 7.06 7.06
5.47 5.46 5.45 5.44 5.44
5.05 5.03 5.02 5.01 5.00 4.98 4.98 4.97
3.81 3.79 3.79 3.77 3.75 3.67 3.65 3.64 3.63
1.27 1.26 1.25 1.25 1.24 1.23 1.22 1.20
0.99 1.00 0.97 1.04 0.95 0.97 1.01 1.03 6.26
f1 (ppm)

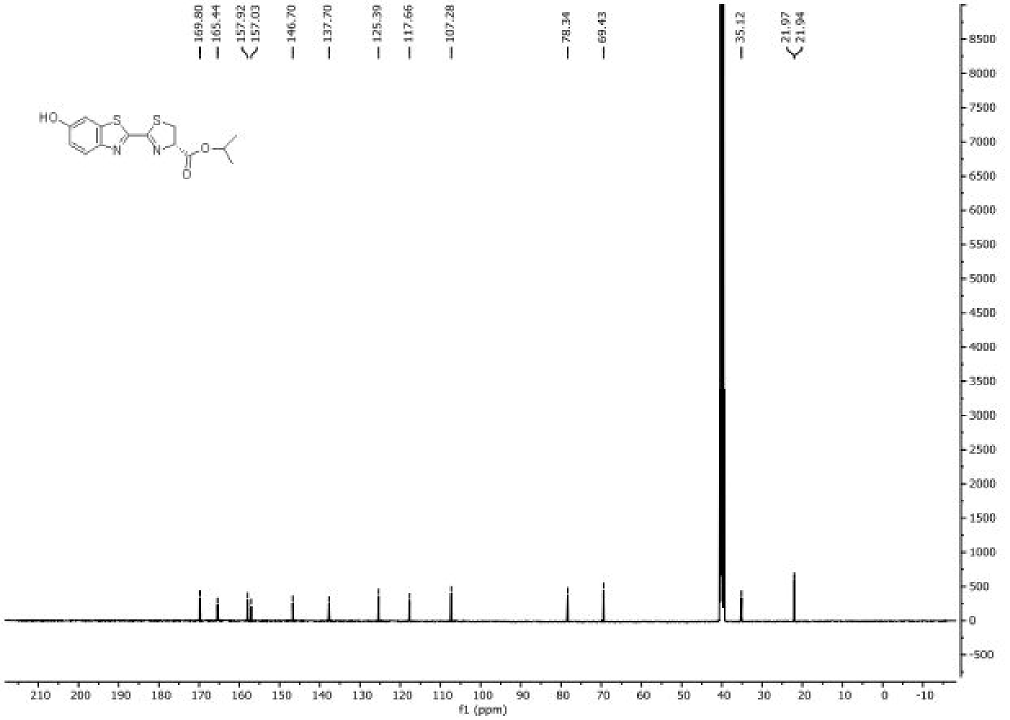
169.80 165.44 157.92 157.03 146.70 137.70 125.39 117.66 107.28 78.34 69.43 35.12 21.97 21.94
f1 (ppm)

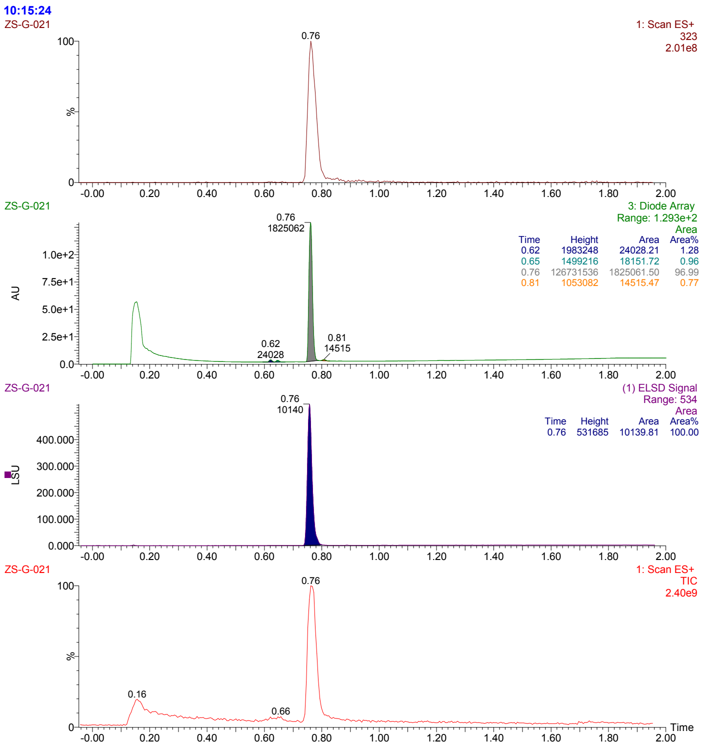

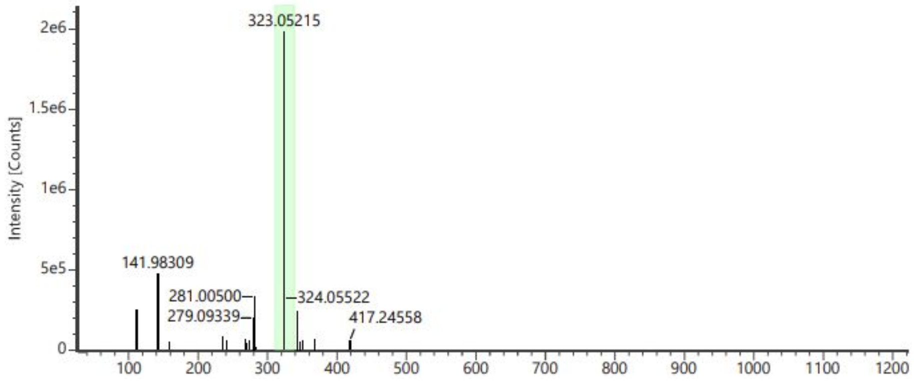

.	Component name	Observed RT (min)	Neutral mass (Da)	Observed m/z	Mass error (mDa)	Mass error (ppm)
1	luciferin	1.89	322.04458	323.0521	0.3	0.9

Figure S1: $^{1}H/^{13}C$ NMR Spectra (DMSO-d_6), UPLC, and HRMS Data for Luciferin Isopropyl Ester.
